# Synthesis and Characterization of Metallo-Supramolecular Polymers Based on Benzodipyrrolidone

**DOI:** 10.3389/fchem.2021.673834

**Published:** 2021-04-28

**Authors:** Zheng Chi, Hao Dong, Ganhui Shi, Pan Liu, Chenchen Ma, Xuegang Chen

**Affiliations:** Key Laboratory of Rubber-Plastic of Ministry of Education (QUST), School of Polymer Science and Engineering, Qingdao University of Science and Technology, Qingdao, China

**Keywords:** terpyridine, metallo-supramolecular polymers, benzodipyrrolidone, narrow energy gap, coordination

## Abstract

A simple route to the preparation of benzodipyrrolidone (BDP) based monomeric building blocks containing 2,2′:6′,2″-terpyridines is reported from a common precursor 4′-(4-pinacolatoboronphenyl)-2,2′:6′,2″-terpyridine via Suzuki coupling reaction. Self-assembly polymerization with ruthenium (II) ions under mild conditions yielded a series of novel metallo-supramolecular polymers with weak donor-acceptor (D-A) structures based on benzodipyrrolidone. The structure of the bridge connected BDP with terpyridine have a significant impact on the wavelength and intensity of the intramolecular charge transfer (ICT) absorption peak. The resulting metallo-polymers exhibited strong double absorption bands around 315 nm and 510 nm involved in π-π^*^ transitions and ICT or metal to ligand charge transfer (MLCT) absorption bands. The forming of D-A structure and coordination with ruthenium (II) ions is favorable to narrow the energy gap and the energy gaps of the resulting metallo-supramolecular polymers are 2.01 and 1.62 eV, respectively.

## Introduction

Based on intermolecular non-covalent interactions, supramolecular chemistry has become one of the most interesting fields in modern chemistry (Lehn, 2002). Supramolecular polymers, resulted from the combination of supramolecular chemistry and polymer science, a new polymers based on monomeric units held together with directional and reversible secondary interactions such as π-π interactions, metal-ligand coordination bonds, hydrogen bonds, host-guest recognitions, and hydrophobic interactions, have become a focus of growing attention among polymer sciences (Brunsveld et al., 2001; Greef et al., 2009; Vukotic and Loeb, 2012; Yan et al., 2012; Zhang et al., 2020; Li et al., 2021). As a subdivision of supramolecular polymers, metallo-polymers contain a variety of metal centers, mainly transition metals and lanthanides, in main chains or side structures (Yang et al., 2015; Mauro et al., 2017; Wang et al., 2019). 2,2′:6′,2″-Terpyridine is a tridentate ligand which could form stable complexes by coordinating with a wide range of transition metal ions in their low oxidation states based on strong metal-lignd (d-π^*^) back donation (Wild et al., 2011). These complexes possess a distorted octahedral geometry and thereby a linear bilateral conformation of ligands can be obtained, which is critical in construction of metallo-supramolecular polymers and well-defined architectures based on the coordination interactions between terpyridine and metal center (Pandey et al., 2016; Winter and Schubert, 2016; Yin et al., 2018).

Introduction of metal centers into main chains not only endow polymers with novel properties based on these hybrid structures of organic and inorganic but enable various novel units in metallo-supramolecular polymers. Yu et al. reported the synthesis of a family of self-aseembed zinc-terpyridyl-based polymers in which some π-conjugated moieties with high luminescence efficiency have introduced and some light-emitting diodes from these metallo-supramolecular polymers have been fabricated (Yu et al., 2003). Stimulated by these, more metallo-supramolecular materials have been synthesized and applied in organic light-emitting diodes (Chen and Lin, 2007; Winter et al., 2009). Some electron-donor and electron-acceptor π-conjugated units have been also introduced into main chains and some novel light-emitting properties were obtained (Schlütter et al., 2010). Further, by fine design for monomeric structures, some novel metallo-supramolecular polymers have been developed with good performances in photovoltaic cells (Vellis et al., 2008; Padhy et al., 2011; Feng et al., 2013).

In recent years, incorporating so-called high-performance pigments such as diketopyrrolopyrrole (DPP), isoindigo (IIG), and quinacridones, used as electron-accepting building blocks, into π-conjugated polymers have been the most frequently way to construct D-A type polymers with narrow band gaps and high charge carrier mobility (Zhang and Tieke, 2014; Zhang et al., 2017, 2018). Among numerous metallo-supramolecular polymers, there seldom has metallo-polymers containing these classic or novel pigments, such as 1,3,4,6-tetraarylpyrrolo[3,2-b]pyrrole-2,5-dione (isoDPP), benzodipyrrolidone (BDP) and naphthodipyrrolidone (NDP) in their backbone skeletons. As a novel electron-withdrawing unit, benzodipyrrolidone (BDP) is similar in structure to DPP, but better planarity, highly conjugated and lactam structures make BDP ideal electron-with drawing unit and promising in construct D-A polymers or n-type polymers with reduced energy gaps (Cui et al., 2011; Cui and Wudl, 2013; Rumer et al., 2013; Kawabata et al., 2016).

Herein, we report the preparation of a serials novel metallo-supramolecular polymers based on benzodipyrrolidone (BDP) via coordination interactions between 2,2′:6′,2″-terpyridines and ruthenium (II) ions. The monomeric building blocks with different electron donating capability and conjugation degree were prepared by means of Suzuki coupling reaction and the metallo-supramolecular polymers can be obtained via self-assembly polymerization of the monomeric building blocks under the inducing of the metal ions (Supplementary Scheme 1). The polymers showed very attractive properties, and full characteristics have been carried out.

## Results and Discussion

**Figure 1:**
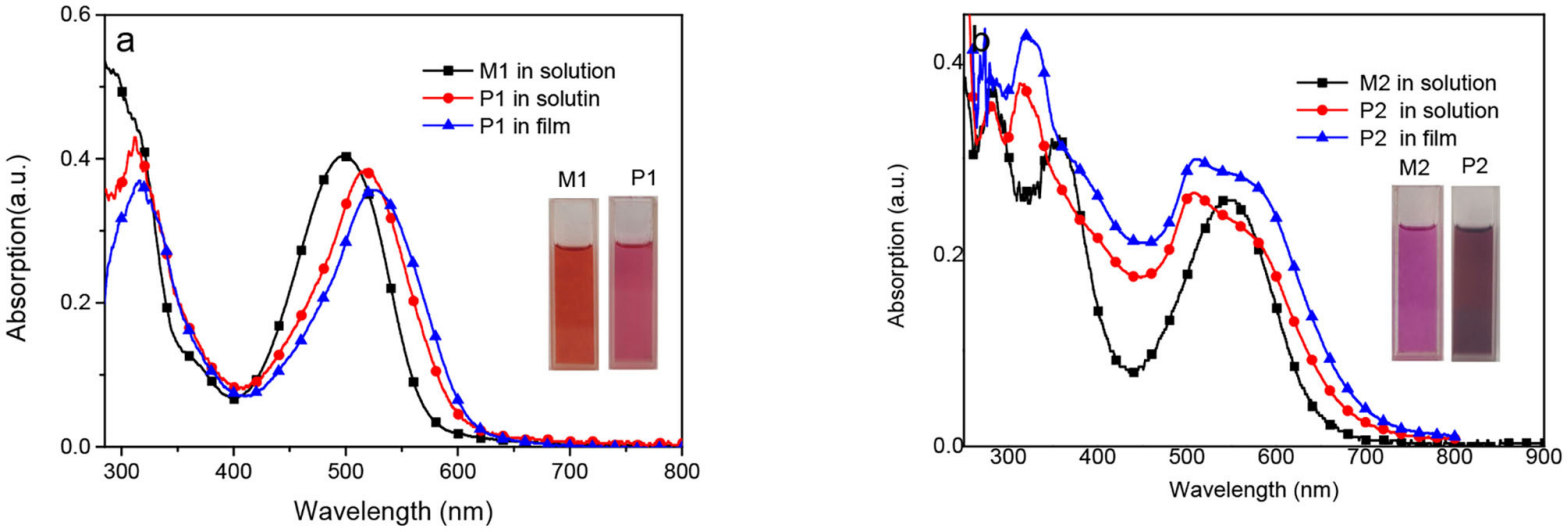
UV-vis absorption spectra of the monomer **M1** and polymer **P1 (a)**, and monomer **M2** and polymer **P2 (b)** in dilute DMAc solution or films.

The details for synthesis and some characteristics data of the monomeric building blocks and the metallo-supramolecular polymers are described in the Supporting Information. UV-Vis absorption spectra of the monomers **M1** and **M2**, and polymers **P1** and **P2** are shown in Figure 1 and summarized in Supplementary Table 1. As shown in Figure 1, **M1** and **M2** in solution present two absorption bands with the absorption maxima at 286, 497, 356, 547 nm, respectively. The lower wavelength absorption can be attributed to the π-π^*^ electronic transitions of the conjugated structures, while the longer wavelength absorption can be attributed to the intramolecular charge transfer transition (ICT) inside the monomers containing electron-withdrawing unit BDP (A) and electron-donating unit (D) phenyl or thiophene rings. In fact, there occur weak D-A structures in building blocks **M1** and **M2**, and resulting metallo-supramolecular polymers **P1** and **P2**. Compared with that of **M1**, the obvious red-shift of the absorption maxima for **M2** is due to the conjugation extension and the stronger ability of electron-donating of thiophene moieties in **M2**. The metallo-supramolecular **P1** and **P2** present similar two absorption bands to that of the monomers. The absorption maxima near 315 nm for **P1** and **P2** arised from π-π^*^ electronic transitions show little change compared with that of monomers **M1** and **M2**. The absorption maxima at low energy bands for **P1** and **P2** near 510 nm can be attributed to the combination of intermolecular charge transfer transition (ICT) and metal to ligand charge transfer (MLCT) (Duprez et al., 2005). Different from **M1** and **M2**, the red-shift and broadening of the absorption band for **P1** and **P2** is partly related to the increased acceptor properties of its π^*^ orbitals upon coordination with ruthenium ion (Roberto et al., 2002) and especially the increased ICT from thiophene unit to Ru(II)-coordinated terpyridine in **P2** leaded to more broad absorption band. An evident shoulder peak at about 580 nm for **P2** derives from a certain degree of π-π stacking of the related backbone even in the solution state, and there is more obvious shoulder peak in film as shown in Figure 1b, which could be favorable to have a higher carrier mobility in device applications. It is interesting that **P2** shows not only broad peaks but also the strong absorption band covering from 300 to 600 nm, which suggest that these novel metallo-supramolecular polymers should be good candidate for high effective light-absorbing materials in organic heterojunction solar cells.

**Figure 2:**
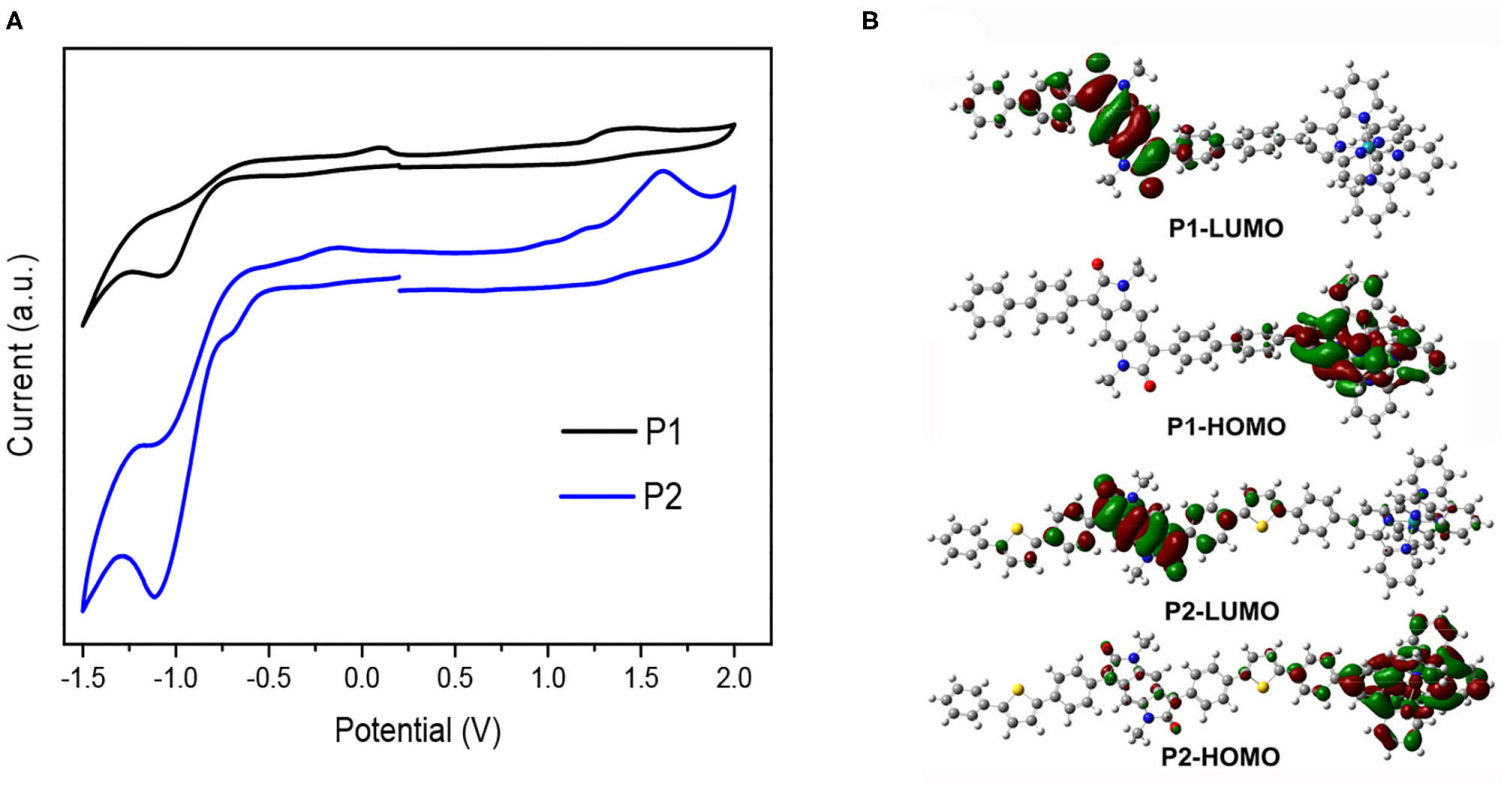
Cyclic voltammograms (CV) curves of the metallo-supramolecular polymers **(A)** and simulated HOMO and LUMO orbitals for the polymers **P1** and **P2** calculated at the B3LYP/6-31(d,p) level **(B)**.

Electrochemical properties of **P1** and **P2** were explored by cyclic voltammetry (CV) measurement. Depicted in Figure 2A is the cyclic voltammograms (CV) of the metallo-supramolecular polymers. The metallo-polymers show quasi-reversible oxidation and reduction during positive and negative scans. The reductive peaks are much stronger than their oxidative counterparts, indicating that these metallo-supramolecular polymers are more easily reduced than oxidized, which is partly due to the strong electron-withdrawing ability of the BDP units and the increasing of electron-deficiency from coordination of terpyridine with metal ions. The HOMO and LUMO energy levels and the energy gap (E_g, CV_) of the polymers were calculated from the onset oxidation potentials (E_onset, ox_) and the onset reduction potentials (E_onset, red_) of the polymers, using a ferrocene/ferrocenium (Fc/Fc^+^) redox couple (4.80 eV below the vacuum level) as an external standard. The HOMO energy levels were estimated to be ca. −5.56 eV for **P1** and −5.40 eV for **P2**, respectively. Similarly, the LUMO energy levels of **P1** and **P2** were estimated to be ca. −3.55 and −3.78 eV, respectively. Compared with **P1** (the energy gap is 2.01 eV), the introducing of electron-donating moiety thiophene into main chain skeleton in **P2** affect both the HOMO and LUMO energy levels of **P2** obviously and thereby the reduced energy gap 1.62 eV for **P2**.

For a better understanding of the electronic properties of the metallo-supramolecular polymers, the molecular orbitals were calculated using density functional theory (DFT) at the B3LYP level using 6-31G(d,p) as a basis set for C, H, N, O, and S atoms and LANL2DZ for Ru. To simplify the calculation, all the alkyl groups were replaced with methyl groups. As shown in Figure 2, the HOMOs of all the ruthenium-containing polymers **P1** and **P2** are delocalized along the conjugated backbones of benzene or thiophene rings and mainly in Ru coordinated terpyridine moieties. This result indicates that the HOMO energy levels can be determined by the donor, and to a large degree, there are markedly MLCT transitions in the resulting metallopolymers. In contrast to the HOMOs, the LUMOs are distributed mainly on the acceptor units (BDP cores and terpyridines moieties). The coordination interactions between the 2,2':6',2"-terpyridine and the transition ion Ru^2+^ also maybe in some degree have some influence on the LUMO energy levels in resulting metallo-supramolecular polymers. This result implies that the LUMO energy levels can be determined by the acceptor unit BDP, and the acceptor unit can be modified to tune the LUMO energy levels precisely. These calculated results are consistent with the experimentally determined results mentioned above.

## Conclusion

Introducing high performance pigments into metallo-polymers, we synthesized and characterized a novel BDP-based metallo-supramolecular polymers via the non-covalent bonds coordination interaction between terpyridine and ruthenium (II) ions in mild condition. Due to the weak D-A structure in main chain came from the strong electron-accepting unit BDP and electron-donating benzene rings or/and thiophene ring, both resulting metallo-supramolecular polymers P1 and P2 showed π-π^*^ transitions absorbtion band around 315 nm and strong and broad ICT and MLCT absorption bands around 510 nm. The conjugated and electron-donating unit in backbone can influence the UV-vis absorption properties, energy levels and thereby energy gaps. Consequently the polymer P2 exhibited narrow energy gap (1.6 eV). All of this suggest that utilizing fine molecular designing and interaction of building blocks and metal ions, the photophysical and electrochemical properties of resulting metallo-polymers can be regulated efficiently, which is a promising method to develop photoelectric materials with high performances.

## Data Availability Statement

The original contributions presented in the study are included in the article/Supplementary Material, further inquiries can be directed to the corresponding author/s.

## Author Contributions

ZC and HD prepared materials and carried out in experiments. GS, PL, and CM helped to analyze experimental data. XC supervised the work and prepared final text and revised. All authors contributed to the article and approved the submitted version.

## Conflict of Interest

The authors declare that the research was conducted in the absence of any commercial or financial relationships that could be construed as a potential conflict of interest.
