# Supplementary material for: Synthesis and Characterization of Metallo-Supramolecular Polymers Based on Benzodipyrrolidone
Source: Front Chem. 2021 Apr 28;9:673834. doi: 10.3389/fchem.2021.673834 (PMC8113679; doi:10.3389/fchem.2021.673834)
Supplement: Supplementary file 1 [file Data_Sheet_1.docx]

**Supporting Information**

**Synthesis and Characteristics of Metallo-supramolecular Polymers based on Benzodipyrrolidone**

Zheng Chi, Hao Dong, Ganhui Shi, Pan Liu, Chenchen Ma, Xuegang Chen^*^

Key Laboratory of Rubber-Plastic of Ministry of Education (QUST), School of Polymer Science and Engineering, Qingdao University of Science and Technology, Qingdao 266042, China

*Correspondence: Xuegang Chen [xgchen@qust.edu.cn](mailto:xgchen@qust.edu.cn);

**Contents**

**Materials and synthesis.**

**Instrument and measurement**

**Scheme S1:** Synthesis of building blocks **M1** and **M2** and Ruthenium(II) supramolecular polymers **P1** and **P2**.

**Table S1:** Properties of the metallo-supramolecular polymers **P1** and **P2**.

Figure S1: ^1^H-NMR and ^13^C-NMR of **M1**

Figure S2: ^1^H-NMR and ^13^C-NMR of **M2**

**References**

**Materials and synthesis**

Tetrahydrofuran (THF) and toluene were dried by sodium and distilled prior to use. The precursor BDP derivative (**1**) (Cui et al., 2011) 4´-(4-pinacolatoboronphenyl) -2,2´:6´,2´´-terpyridine (**4**) (Aspley et al., 2001) were synthesized according to the literature. Other chemicals were purchased from commercial resources and used as received.

## Synthesis of compound 2

To a solution of **1** (0.17 g, 0.16 mmol) and tributyl(thiophen-2-yl)stannane (0.12 g, 0.33mmol) in toluene (10 mL) was added Pd(PPh_3_)_4_ (0.02 g, 0.017 mmol) at room temperature. The mixture was stirred at 110 ^o^C for 24 h. The resulting mixture was extracted with chloroform and the organic extracts were washed with brine three times and dried with MgSO_4_ and concentrated in a vacuum. The residue was purified via column chromatography on silica gel with petroleum ether (PE):dichloromethane (DCM) (1:2, v/v) as eluent to give compound **2** as purple solid (0.15 g, 88%). ^1^H NMR (500MHz, CDCl_3_): δ (ppm) 7.76 (d, J = 5.0 Hz, 4H), 7.72 (d, J = 5.0 Hz, 4H), 7.39 (d, J = 5.0 Hz, 2H), 7.36 (d, J = 5.0 Hz, 2H), 7.11 (t, J = 7.5 Hz, 2H), 6.41 (s, 2H), 3.43 (t, J = 7.5 Hz, 4H), 1.64 (t, J = 6.3 Hz, 2H), 1.58-1.22 (m, 64H), 0.88 (t, J = 7.5 Hz, 12H).

Synthesis of compound **3**

To a solution of **2** (150 mg, 0.14 mmol) in THF (15 mL) was added N-Bromosuccinimide (NBS) (53 mg, 0.30 mmol) at room temperature and the mixture was stirred at ambient temperature for 19 h. The resulting mixture was extracted with chloroform and the organic extracts were washed with brine three times and dried with MgSO_4_ and concentrated in a vacuum. The residue was purified via column chromatography on silica gel with petroleum ether (PE):dichloromethane (DCM) (1:1, v/v) as eluent to give compound **3** as deep purple solid (97 mg, 57%). ^1^H NMR (500MHz, CDCl_3_): δ (ppm) 7.75 (d, J = 5.0 Hz, 4H), 7.62 (d, J = 5.0 Hz, 4H), 7.14 (d, J =5.0 Hz, 2H), 7.06 (d, J = 5.0 Hz, 2H), 6.38 (s, 2H), 3.52 (t, J = 7.5 Hz, 4H), 1.78 (m, 2H), 1.56-1.22 (m, 64H), 0.87 (t, J = 7.5 Hz, 12H).

Synthesis of compound **M1**

To a mixture suspension of compound **1** (0.22g, 0.21mmol), compound **4** (0.19g, 0.43mmol), Na_2_CO_3_ (0.16g, 1.28mmol) in 0.9 mL H_2_O were added Pd(dppf)Cl_2_ (19.2 mg, 0.03mmol) and THF (8.9 mL). The mixture was heated to 72 ^o^C and stirred for 36 h. The resulting mixture was extracted with chloroform and the organic extracts were washed with brine three times and dried with MgSO_4_ and concentrated in a vacuum. The residue was purified via column chromatography on silica gel with petroleum ether (PE):dichloromethane (DCM) (1:2, v/v) as eluent to give compound **M1** as deep red solid (0.13 g, 41%). ^1^H NMR (500MHz, CDCl_3_): δ (ppm) 8.82 (s, 4H), 8.74 (d, J = 5.0 Hz, 4H), 8.69 (d, J = 7.5 Hz, 4H), 8.04 (d, J = 7.5 Hz, 4H), 7.92-7.86 (m, 8H), 7.82-7.80 (m, 8H), 7.39-7.37 (m, 4H), 6.47 (s, 2H), 3.57 (t, J = 7.5 Hz, 4H), 1.81 (m, 2H), 1.56-1.22 (m, 64H), 0.85 (m, 12H)。 ^13^C NMR (500MHz, CDCl_3_): δ ppm 169.97, 156.30, 156.09, 149.65, 149.18, 144.29, 140.90, 137.91, 136.90, 134.33, 130.80, 130.11, 127.91, 127.54, 126.84, 123.87, 121.41, 118.75, 97.52, 44.13, 37.20, 31.91, 30.07, 29.68, 29.37, 26.77, 22.68, 14.09. Anal. calc. for C_104_H_120_N_8_O_2_: C, 82.50; H, 7.99; N, 7.40 Found: C, 83.11; H, 7.86; N, 7.55.

Synthesis of compound **M2**

**M2** was synthesized from compound **3** and compound **4** following the same procedure for the synthesis of **M1**.Yield: 73% (0.11 g). ^1^H NMR (500MHz, CDCl_3_): δ (ppm) 8.78 (s, 4H), 8.73 (d, J=5.0Hz, 4H), 8.68 (d, J=7.5Hz, 4H), 7.98-7.78 (m, 18H), 7.43-7.35 (m, 10H), 6.43 (s, 2H), 3.55 (t, J=7.5Hz, 4H), 1.82 (m, 2H), 1.54-1.25 (m, 64H), 0.85 (m, 12H)。 ^13^C NMR (500MHz, CDCl_3_) δ ppm 169.89, 167.70, 156.29, 156.05, 149.41, 149.16, 144.17, 143.78, 143.38, 137.63, 136.86, 134.85, 134.01, 132.50, 132.40, 130.86, 130.10, 128.84, 127.88, 126.03, 125.72, 124.89, 124.79, 123.83, 121.39, 118.53, 97.51, 44.05, 36.79, 31.92, 30.06, 29.69, 29.36, 26.83, 22.68, 14.04. Anal. calc. for C_112_H_124_N_8_O_2_S_2_: C, 80.15; H, 7.45; N, 6.68 Found: C, 80.70; H, 7.16; N, 6.74.

General process for synthesis of polymers

To the mixture of AgBF_4_ ( 44.3 mg, 0.23 mmol), RuCl_3_.xH_2_O (17.9 mg, 0.07 mmol) was added dried acetone (6 mL) and refluxed for 2 h. The resulting suspension was filtered and the filtrate was evaporated and the resulting solid was added to the solution of **M1** (0.10g, 0.07mmol) in *n*-butyl alcohol (20 mL). The mixture was refluxed and stirred for 3 days. The resulting solution was pour into ice water (about 150 mL) and the precipitate was filtered, washed with cold methanol and finally dried under vacuum. The polymer **P1** was obtained as a black solid (95 mg, 83%). ^1^H NMR (500MHz, D7-DMF) δ ppm 9.36-9.25 (br), 8.85-8.75 (br), 8.40-8.37 (br), 8.30-8.22 (br), 8.16 -7.38 (br), 6.45-6.42 (br), 3.68-3.02 (br), 2.46-0.52 (br).

The polymer **P2** as purple black solid was obtained from **M2** with similar procedure. Yied: 89%. ^1^H NMR (500MHz, D7-DMF): δ ppm 9.38-9.27 (br), 8.74-8.66 (br), 8.53-8.36 (br), 8.25-7.36 (br), 6.43-6.40 (br), 3.72-3.11 (br), 2.58-0.44 (br).

**Instrument and measurement**

^1^ H NMR and ^13^C NMR spectra were recorded on a Bruker Avance 500 MHz spectrometer and tetramethylsilane (TMS) as an internal reference. Elemental analysis was performed with a VarioEL analysis system. The optical properties were measured with Hitachi U-4100 UV-vis spectrometer and the polymer films were prepared from spin-coating process. Electrochemical cyclic voltammetry (CV) (Metrohm Autolab PGSTAT204 analyzer) was conducted with a platinum working electrode, a platinum wire counter electrode, and an Ag wire quasi-reference electrode in anhydrous acetonitrile using tetrabutylammonium perchlorate as the supporting electrolyte at a potential scan rate of 50 mV/s.

Scheme S1 Synthesis of building blocks **M1** and **M2** and Ruthenium(II) supramolecular polymers **P1** and **P2**.

Table S1 Properties of the metallo-supramolecular polymers **P1** and **P2**.

| Monomer/Polymer | η_inh_^a^ (dL g^−1^) | λ_abs,max_^b^ | | Energy levels ^c^ (eV) | | E_g,CV_(eV) |
| --- | --- | --- | --- | --- | --- | --- |
|  |  | Solution | Film | HOMO | LUMO |  |
| **P1** | 1.46 | 312, 517 | 315, 527 | -5.56 | -3.55 | 2.01 |
| **P2** | 1.68 | 315, 507, 582(sh) | 319, 508, 584(sh) | -5.40 | -3.78 | 1.62 |

^a^ Inherent viscosity measured in NMP at 30 ◦C using a Ubbelohde viscometer.

^b^ Solutions in DMAc dilute solution (2 × 10^−5^ mol L^−1^), and thin films spin-coated from DMAc solutions on glass substrates.

^c^ HOMO/LUMO level was calculated from measured oxidation/reduction potential in DMAc solution (2 × 10^−3^ mol L^−1^) and energy gap was calculated according to E_g,CV_= LUMO-HOMO.


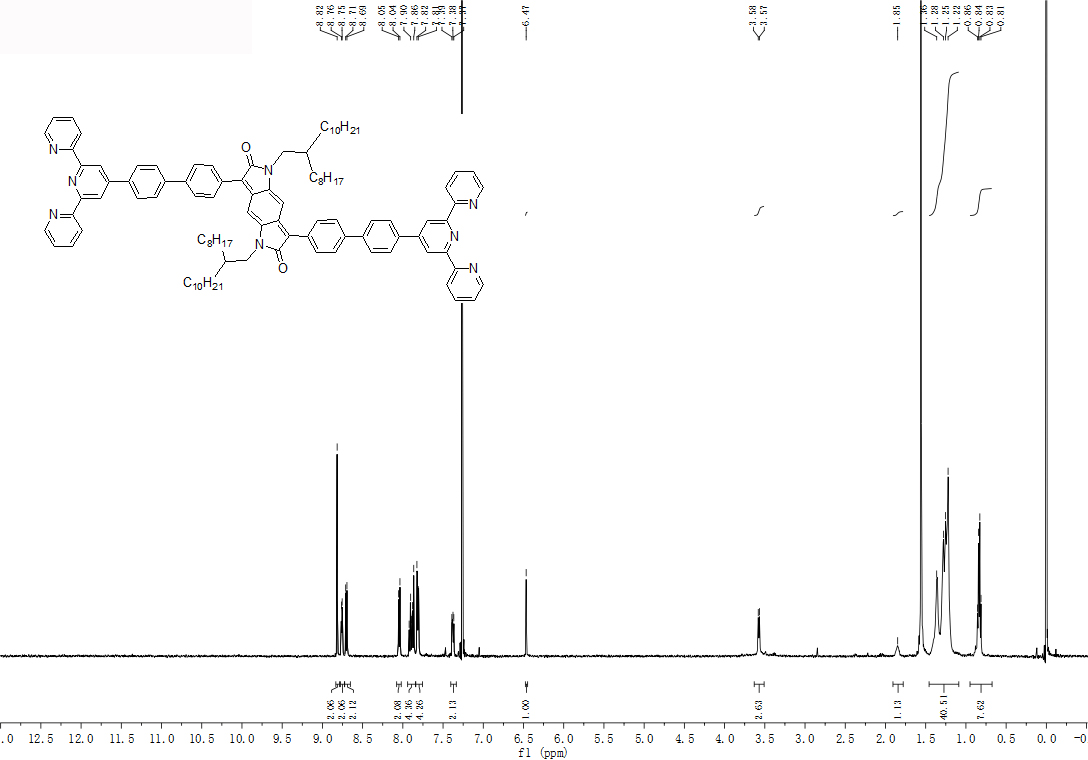


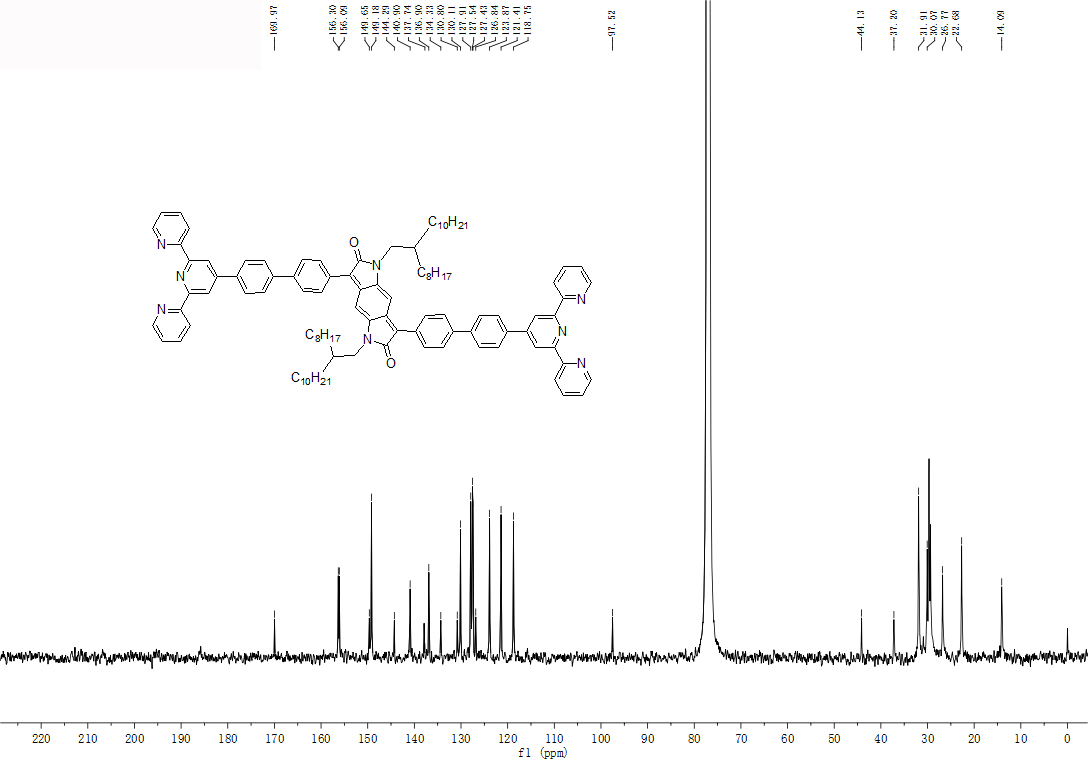


Figure S1: ^1^H-NMR and ^13^C-NMR of **M1**


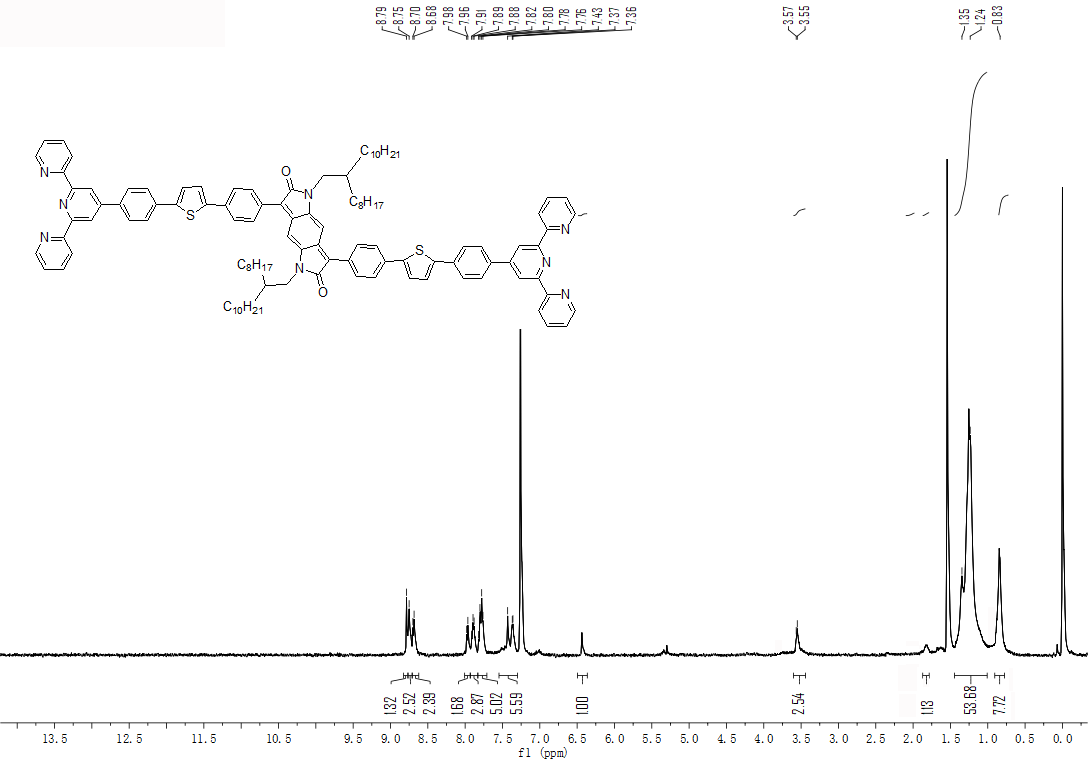


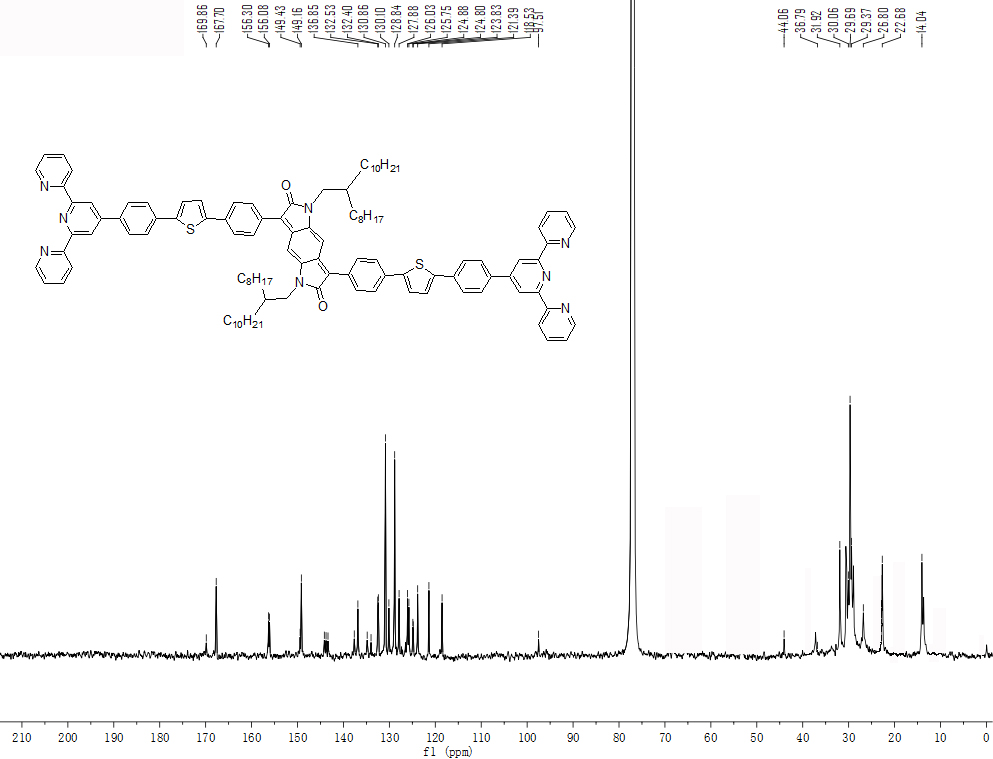


Figure S2: ^1^H-NMR and ^13^C-NMR of **M2**

**References**

Cui, W., Yuen, J., and Wudl, F. (2011) Benzodipyrrolidones and their polymers. *Macromolecules* 44, 7869-7873. doi: 10.1021/ma2017293

Aspley, C. J. and Williams J. A. G. (2001). Palladium-catalysed cross-coupling reactions of ruthenium bis-terpyridyl complexes: strategies for the incorporation and exploitation of boronic acid functionality. ***New J. Chem.* 25**, 1136-1147. doi: [10.1039/B103062K](https://doi.org/10.1039/B103062K)
